# Evaluating two implementation approaches to support a nationally mandated caregiver skills training in veterans affairs: a type III hybrid implementation-effectiveness randomized controlled trial

**DOI:** 10.1186/s43058-026-00960-9

**Published:** 2026-06-11

**Authors:** Courtney H. Van Houtven, Kasey Decosimo, Cynthia J. Coffman, Swetha Kota, Connor Drake, Nina R. Sperber, Jaime M. Hughes, Leah L. Zullig, Matthew Tucker, Amy Webster, Emily Franzosa, Amanda Blok, Trisha Chadduck, Leah Christensen, Brystana G. Kaufman, Megan Shepherd-Banigan, Kelli D. Allen, Susan Nicole Hastings

**Affiliations:** 1 Center of Innovation to Accelerate Discovery and Practice Transformation, Durham VA Health Care System, 508 Fulton Street, Durham, NC 27705 USA; 2 Department of Population Health Sciences, Duke University School of Medicine, Durham, NC USA; 3 Duke-Margolis Institute for Health Policy, Duke University, Durham, NC USA; 4 Department of Biostatistics and Bioinformatics, Duke University School of Medicine, Durham, NC USA; 5 Department of Implementation Science, Wake Forest School of Medicine, Winston-Salem, NC USA; 6 Section on Gerontology and Geriatric Medicine, Division of Internal Medicine, Wake Forest School of Medicine, Winston-Salem, NC USA; 7 Geriatric Research, Education and Clinical Center, James J. Peters VA Medical Center, Bronx, New York USA; 8 Brookdale Department of Geriatrics and Palliative Medicine, Icahn School of Medicine at Mount Sinai, New York, USA; 9 Center for Clinical Management Research, VA Ann Arbor Healthcare System, Ann Arbor, MI USA; 10 Department of Systems, Populations and Leadership, University of Michigan School of Nursing, Ann Arbor, MI USA; 11 Veteran’s Health Administration, Washington, DC USA; 12 Department of Medicine & Thurston Arthritis Research Center, University of North Carolina at Chapel Hill, Chapel Hill, NC USA; 13 Center for the Study of Aging and Human Development, Duke University School of Medicine, Durham, NC USA; 14 Geriatric Research, Education, and Clinical Center, Durham VA Health Care System, Durham, NC USA; 15 Division of General Internal Medicine, Department of Medicine, Duke University School of Medicine, Durham, NC USA

**Keywords:** Family caregivers, Veterans, Skills training, Informal care, Implementation science

## Abstract

**Background:**

Few caregiver support interventions have been scaled nationally; thus, gaps remain regarding the optimal level of support needed to successfully adopt caregiver trainings in diverse clinical contexts. We evaluated the effectiveness of two implementation support approaches on implementation outcomes for a family caregiver training (Caregivers FIRST) in partnership with the Veterans Health Administration Caregiver Support Program (CSP).

**Methods:**

In a hybrid type III effectiveness-implementation trial, we enrolled 25 non-adopter sites six months following the national mandate Caregivers FIRST to compare two different implementation support approaches (foundational support or foundational support with the addition of group external facilitation (enhanced support)). Co-primary site-level penetration outcomes at 12-months were 1) percentage of caregivers attending ≥ 1 class among those with any caregiver referral or enrolled in CSP and 2) number of classes delivered. Secondary outcomes were fidelity and adoption. Fidelity was proportion of expected classes delivered of those promised; adoption was delivering at least one round of classes to 5 or more caregivers. Secondary analysis examined outcomes at six and 18-months separately. We fit generalized linear models to examine differences in outcomes between arms. Survey, interview, and administrative data were used to contextualize findings and assess implementation support costs.

**Results:**

Twelve sites were randomized to foundational support and 13 to enhanced support. At 12-months, the estimated mean percentage of qualifying caregivers attending ≥ 1 class was 8.3% and mean number of classes offered was 9.4 for enhanced support and in foundational support were 6.1% and 5.4, respectively. No differences were found by arm at 12-months in mean percentage of caregivers attending a class (mean difference = 2.2%; 95% CI −1.8%,6.1%; *p* = 0.26) or mean number of classes offered (rate ratio = 1.7; 95% CI 0.9,3.4; *p* = 0.11). Neither fidelity nor adoption were different by arm at 12-months, and similar patterns were found at additional timepoints. The added cost of delivering enhanced support per site was $3,678 over a one-year period (median foundational =$14,985 vs. enhanced =$18,663).

**Conclusions:**

Foundational support allowed most sites (75% foundational, 100% enhanced) to successfully adopt by 18 months. While not reaching a priori benchmarks in magnitude, observed means of implementation outcomes at enhanced support sites were higher at 12 months.

**Trial registration:**

This study was registered on April 8, 2022 at ClinicalTrials.gov (identifier NCT05319535). https://clinicaltrials.gov/ct2/show/NCT05319535.

**Supplementary Information:**

The online version contains supplementary material available at 10.1186/s43058-026-00960-9.

## Contributions to the literature

• We demonstrate how foundational and enhanced implementation support change penetration, fidelity, adoption, and costs when offered to sites unable to meet initial adoption benchmarks for delivering a mandated caregiver training, Caregivers FIRST.

• To date in the United States, few caregiver trainings have been implemented at scale in clinical practice. We used two robust, standardized implementation support approaches based on a framework that promoted adaptations and tailoring for context.

• Health systems considering scaling evidence-based caregiver trainings can form realistic expectations about implementation outcomes from our study and evaluate (lack of) differences in implementation outcomes in foundational and enhanced support approaches.

## Background

Momentum is building nationally to incentivize and expand family caregiver training and supports in the United States, following formulation of the National Caregiver Strategy that proposed scaling of evidence-based trainings as a key tenet [1]. Independently but consistent with this call for expansion of training, the Centers for Medicare and Medicaid Services (CMS) established new reimbursement mechanisms in 2024 to support the delivery of family caregiver training programs and introduced the ‘Guiding an Improved Dementia Experience’ (GUIDE) Model in 2023 [2], intended to support people living with dementia and their unpaid caregivers.

Since health systems, group practices, and providers now can be reimbursed for group caregiver trainings, they must decide what type, size, and duration of trainings to offer within the new reimbursement structure. Additional decisions to consider are how to match different trainings to the appropriate caregiver populations and how best to implement group training. And yet, in the United States, few evidence-based caregiver support interventions have transcended the research setting to be scaled for widespread dissemination. One approach to addressing implementation barriers is to test the impact of implementation support strategies on reach and integration of new practices into existing systems of care [3].

Caregivers FIRST (Caregivers Finding Important Resources, Support, and Training) is an evidence-based group skills training curriculum for family caregivers that originated as a randomized controlled trial [4] and was announced for national dissemination in partnership with the Veterans Health Administration (VHA) national Caregiver Support Program (CSP) [5]. The VHA is the largest public integrated health system in the country, and has built a comprehensive CSP with services and supports (e.g., training, mentoring, coaching, referrals) for family caregivers of Veterans using VHA [6]. Once Caregivers FIRST was announced as a part of CSP’s suite of services, it was delivered using Replicating Effective Programs (REP), a bundle of implementation strategies consisting of technical assistance and external facilitation in an eight-site trial [7, 8]. The evaluation highlighted that participating sites required varying support to successfully implement the program, ranging from minimal technical assistance to intensive assistance and support [5]. Subsequently in 2022, CSP announced Caregivers FIRST as a mandatory group training included in a site’s performance evaluation. Despite the time-sensitive need for caregiver training in real-world settings, there is a limited understanding of the optimal level of implementation support and facilitation required to adopt and sustain a caregiver training program as part of routine care patterns.

This paper presents results of 25 VHA sites unable to meet Caregivers FIRST adoption benchmarks in a hybrid effectiveness implementation cluster randomized trial comparing implementation outcomes at 12 months between sites receiving foundational implementation support only versus foundational support plus the addition of enhanced support consisting of group external facilitation. Implementation outcomes of interest were also evaluated at six and 18 months to understand the dynamic implementation process. Building upon prior work that evaluated clinical effectiveness [8] and a team collaboration strategy on implementation [9], we hypothesized that enhanced implementation support would lead to a significant increase in implementation penetration, fidelity, and adoption of Caregivers FIRST compared to foundational support alone. The study has four objectives in reporting the primary focus of this hybrid type III design (implementation outcomes): (1) describe baseline organizational contextual factors for randomized sites and the relationship between organizational contextual factors and implementation outcomes, (2) evaluate implementation outcomes (penetration, fidelity, adoption) at 12 months (primary) and 18 months, (3) describe implementation contextual factors qualitatively, and (4) report the estimated delivery costs of the two support bundles. Lessons learned can help inform other health systems about how best to scale caregiver trainings.

## Methods

Caregivers FIRST is one of three evidence-based programs evaluated under the Optimizing Function and Independence Quality Enhancement Research Initiative (Function QUERI) [10]. This study was approved as exempt research by the Institutional Review Board (Durham VA Healthcare System) and registered at Clinical Trials (NCT05319535). Caregivers FIRST was considered part of clinical care at participating sites; therefore, no consent was required for analysis of implementation outcomes extracted from the electronic health record (EHR). VHA providers gave consent for survey and qualitative interviews participation. This study is reported following the Standards for Reporting Implementation Studies (StaRI) checklist (Additional file 1).

### Overview

Using a hybrid type III effectiveness-implementation trial, sites unable to meet Caregivers FIRST adoption benchmarks within six months following the announcement of the program as mandated within CSP (October 2021) were eligible. As Caregivers FIRST scaled nationally, this trial design was selected to test our primary hypothesis that sites randomized to enhanced implementation support would have greater implementation success compared to those randomized to foundational implementation support. Adoption benchmarks were defined as no Caregivers FIRST activity or less than five unique caregivers trained (a threshold determined in partnership with CSP as ‘non-adopters’) [5]. Sites were identified for recruitment via a program dashboard developed jointly with CSP that used electronic health record documentation to monitor site implementation of caregiver training. Sites were prioritized for recruitment to achieve variation in VHA service region, facility complexity level, prior Caregivers FIRST implementation activity, and total CSP staffing capacity. Eight sites that delivered Caregivers FIRST as part of the previous Function QUERI trial [8] were excluded.

Enrolled sites were randomized into two implementation support arms using a stratified block randomization (block size of two) and two dichotomous stratification variables: 1) Facility complexity was coded as high complexity versus low using a VHA rating of hospitals based on levels of patient volume and risk, teaching and research, intensive care units, and physician specialist staffing [11]. High complexity hospitals were ‘1a’, ‘1b’ or ‘1c’ while low complexity hospitals were ‘2’, ‘3’, and ‘no complexity’; and 2) prior implementation of Caregivers FIRST before the mandate announcement (e.g., implemented in fiscal year 2021 or 2022 vs. none). The first arm included foundational support only and the second arm included foundational support plus enhanced support. Clinical delivery staff and study staff were not blinded to randomization arm assigned. Surveys and interviews were conducted with site staff to understand program implementation context facilitators, barriers, strategies related to variation in program implementation outcomes. Enrollment for all sites began on April 25, 2022, with 12-month follow-up through April 24, 2023, and 18-month follow-up ending October 23, 2023.

### Caregivers FIRST intervention

As described elsewhere [5], Caregivers FIRST is a group training curriculum for family and friend caregivers of Veterans with functional limitations that originated from a randomized controlled trial and has been pragmatically adapted for feasible adoption into usual clinical practice. Caregivers FIRST is designed to address caregiver coping, support seeking, hands-on (e.g., transfers, mobility, communication), and health system navigation skills [11]. The training includes four core sessions (approximately 60–75 minutes) as well as six optional topics delivered by clinical staff within CSP or partnered with other service lines (e.g., chaplaincy, mental health, or primary care) [5]. Caregivers FIRST is unique in that it can target caregivers of Veterans of any diagnosis and can be delivered in person or virtually.

### Implementation approaches

*Implementation frameworks.* We constructed an overarching implementation framework [5] for conducting and designing implementation strategy activities drawing from the Dynamic Sustainability Framework [12] and QUERI Implementation Roadmap [13]. The Dynamic Sustainability Framework model posits that implementation is a dynamic and iterative process that depends on factors related to intervention characteristics and how they interact with a clinical site environmental context, processes to support implementation efforts, and the implementing teams’ capacity. In this case, how the characteristics of Caregivers FIRST interact with the environment and site-based implementation efforts and team at each site. The QUERI Implementation Roadmap posits that these factors occur across distinct stages (pre-implementation; implementation; sustainment) which further shape how they interact to facilitate or obstruct implementation progress. Consistent with our framework that greater implementation success is achieved through rigorous and regular attention to several influencing factors including the characteristics of the intervention itself, we expect that sites with a high readiness to change, resilience ethos, and a climate that prioritizes successful implementation of evidence-based practices will have greater implementation success. However, because of the variation of site contexts (e.g., geography, facility complexity) and the unique needs and circumstances of the implementing teams (e.g., organizational readiness, implementation climate), we propose that providing a low intensity implementation support package (foundational support) that promotes intervention adaptation for context and provides tools for ongoing implementation and evaluation will be sufficient for many sites to successfully adopt Caregivers FIRST. Furthermore, we also posit that adding a more intensive bundle of strategies (enhanced support) specifically focused on practice facilitation, an evidence-based implementation strategy of interactive problem-solving designed to improve team function and readiness for change [14], will be needed to optimize program penetration, fidelity, and adoption [5].

*Foundational implementation support.* Immediately after the national mandate was announced and six months prior to study start, all sites nationally received a low intensity support package consisting of self-guided tools and interactive resources. Foundational implementation support is based on Replicating Effective Programs (REP), a framework focused on enhancing the fit of an evidence-based practice to local contexts while maintaining fidelity to the core components) [5, 10, 15]. Each site received a “welcome call” that detailed components in the foundational support package including access to 1) an implementation toolkit, 2) a SharePoint site with the training curriculum materials and implementation training, 3) a Caregivers FIRST data dashboard to track program delivery, 4) learning collaborative calls led by study staff with study sites to share implementation progress, share best practices, and answer questions, and 5) ongoing technical support and peer mentoring via Microsoft TEAMs channels.

*Enhanced implementation support.* Sites randomized to the enhanced support arm received four 60–90-minute external group facilitation sessions in addition to standard foundational support. These sessions were led by trained study staff over twelve weeks. The session content was based on principles of external facilitation, an implementation strategy rooted in collaborative problem-solving to identify implementation barriers and workshop potential implementation strategies (i.e., tools or activities) to overcome such barriers [14–17]. Sites randomized to enhanced support completed an online needs assessment to assess their implementation status, motivation for adopting Caregivers FIRST, and identification of barriers and facilitators. Call structure and content developed by the study team was informed by the needs assessment and refined prior to each call. Additional details of enhanced implementation support, including its development and delivery, are described elsewhere [18].

### Implementation outcomes

Following the taxonomy defined by Proctor and colleagues [19] and the goals of CSP, we focused on three types of implementation outcomes: penetration (primary), fidelity, and adoption. All implementation outcomes are reported at the site-level, and were assessed at six, 12 (primary), and 18-months. The co-primary outcomes for penetration reflecting the integration of Caregivers FIRST into routine care were: 1) the percentage of caregivers attending a Caregivers FIRST class among all Veterans with referrals to caregiver services or enrolled in CSP and 2) number of unique Caregivers FIRST classes offered. We selected these co-primary outcomes because they are critical metrics of programmatic traction to ensure that Caregivers FIRST is reaching enough Veterans. Secondary implementation outcomes were fidelity and adoption. Fidelity outcomes reflecting the extent to which Caregivers FIRST was delivered as designed were: 1) the percentage of number of recommended classes delivered over the time period (four classes over six months, eight over 12-months and 12 over 18 months); as part of the CSP minimum standard sites were expected to deliver at least 8 classes per fiscal year, 2) the number of caregivers who attended at least one Caregivers FIRST class, and 3) the mean number of classes attended per caregiver. Adoption, a binary outcome reflecting Caregivers FIRST uptake, was defined as delivering a minimum of four training classes to at least five caregivers (Yes) or not meeting this threshold (No). We selected these secondary outcomes with our operational partners to reflect the potential for Caregivers FIRST to be adopted and sustained by the VHA nationally as intended, similarly, thresholds for adoption were defined in consideration of scholarly literature and in coordination with operational shareholders with a lens toward considering what level of activity would be needed for a site to decide to institutionalize Caregivers FIRST.

### Site characteristics

Site characteristics were ascertained at the beginning of the study from VHA administrative reports, including region [20], facility complexity [21], rural designation [22], and CSP staffing and demand for caregiver support services, with the expectation that they may be associated with implementation outcomes. Staff baseline surveys collected validated measures to assess contextual factors that have been used to predict implementation success, including Organizational Readiness to Implement Change (ORIC) short form (9 items) which assesses a site’s commitment to change and efficacy for change [23–25], organizational resilience (the 8-item benchmark resilience tool) which assesses a site’s ability to adapt and change to overcome a new or unexpected change in inner or outer context [26], and implementation climate which assesses a site’s focus, educational support, recognition, rewards, selection for the evidence-based practice, and the selection for openness [27].

### Data collection and measures

Several sources of primary and secondary data were used to evaluate and contextualize Caregivers FIRST implementation: VHA electronic health record data for implementation outcomes, quantitative surveys using Veterans Affairs REDCap (Research Electronic Data Capture) [28], and semi-structured interviews with staff delivering Caregivers FIRST.

Electronic Health Record. Implementation outcomes were assessed from structured text fields in the EHR generated from a national Caregivers FIRST documentation note (included in the CSP mandate), referrals to caregiver services and support, and approval documentation for enrollment in the CSP.

Surveys. For each enrolled site, staff were identified as implementation team members or relevant service line leaders by site-level Points of Contact and were recruited to participate in a baseline survey about factors influencing Caregivers FIRST implementation (Additional file 2). For cost estimates, Points of Contact were asked to report their time and estimate their team’s time related to planning and supporting Caregivers FIRST implementation and other resources used at baseline, six months, and 12 months (Additional file 3).

Implementation activity logs. Study staff collected implementation activity logs including: site attendance and time for implementation support, time spent by the external facilitation team (study staff) on session preparation, delivering implementation support, and follow up support activities (debriefs, documentation), and the estimated time it would take to deliver Caregivers FIRST at a new health system site (e.g., excluding research time).

Qualitative interviews. Semi-structured interviews with Caregivers FIRST implementation team and service line leaders were conducted at 6 months post implementation with a purposive sample of 12 of the enrolled sites, selected to maximize geographic diversity and reported implementation experience (Additional file 4). Semi–structured interviews were developed using inner setting Consolidated Framework for Implementation Research [29] constructs to elicit information about facilitators and barriers to implementation and strategies they used to overcome barriers (Additional file 5); For sites randomized to receive enhanced support, the interview guide also included questions about their experience with enhanced support. All interviews were conducted virtually on the Teams platform.

## Analysis

*Statistical analysis of implementation outcomes*. In following the type III effectiveness-implementation hybrid design, our primary research question compared differences in implementation outcomes (penetration, fidelity, and adoption) between arms at 12 months. Based on a two-sided t-test and a type-1 error rate of 5%, a sample size of 24 sites (randomized 1:1) yielded 80% power to detect an effect size difference of 1.2 (a large effect size difference) and 90% power to detect an effect size difference of 1.4 between arms. Power was not adjusted for co-primary outcomes as success is assessed based on improvement in both measurements of penetration.

Generalized linear models were fit to examine the effect of foundational versus enhanced support on implementation outcomes at six, 12 (primary), and 18 months separately. Models included the arm indicator variable in addition to the two stratification variables used in randomization (high complexity site vs. not); prior implementation of Caregivers First vs. not. For the percentage of caregivers that attended at least one class (penetration), and percentage of number of recommended classes delivered and the mean number of classes attended per caregiver (fidelity), linear regression models were used; for number of classes delivered (penetration) and number of caregivers who attended at least one class (fidelity), a negative binomial regression with a log link were fit; and for adoption, at least four classes delivered to four caregivers, a logistic regression model was used. For linear regression models residuals were assessed for normality assumption. Due to a small sample size and the potential for overfitting, modeling the adoption outcome included only the indicator variable for randomization arm at the 12-month time point. Analyses were performed using SAS 9.4.

*Statistical analysis of survey data.* Descriptive analyses (means, frequencies, and proportions) were conducted for baseline staff survey measures. Mean scores and standard deviations (SDs) were calculated within each site for ORIC short form (9 out of 12 items), organizational resilience (8 items), and implementation climate scale, and then averaged across foundational and enhanced arms.

*Qualitative analysis of interview data.* Interview transcripts were coded and analyzed by two independent coders (MT, AW [30]. Using directed content analysis [31], a priori codes based on the CFIR and Expert Recommendations for Implementing Change (ERIC) typology were used to mark facilitators, barriers, and implementation strategies and activities that participants described; The ERIC implementation strategies were additionally coded by implementation strategy clusters [32]. The qualitative data were then integrated with the 12-month implementation outcomes using a framework matrix as a joint display to support mixed-methods meta inference. The matrix was used to summarize and compare coded site-level qualitative data about facilitators, barriers, and implementation strategies by outcome (fidelity, penetration, and adoption) scores for each arm (foundational, enhanced) [33].

*Budget Impact Analysis of implementation support strategies.* Using implementation activity logs and site-reported time related to planning and supporting Caregivers FIRST (excluding delivery), we conducted a budget impact analysis (BIA) [34–37] to inform the business case by estimating the cost of implementation support strategy bundles for health systems seeking to initiate caregiver training programs [38]. The goal of this approach was to address the immediate question of cost to decision makers who may want to adequately fund implementation support. Direct costs were annualized to cover the 12-month active implementation period (presented in 2022 non-inflation adjusted U.S. dollars). Direct costs included labor for site personnel (e.g., planning, training, supplies) as well as from off-site implementation support (e.g., group external facilitation). For the observations missing a time estimate (*n* = 17%), the median estimate of hours from the non-missing sites were applied within that time period and arm. Development of implementation strategies and materials designed to support Caregivers FIRST implementation were not included in cost estimates and were treated as a sunk cost. Site personnel wages were estimated using 2022 location adjustment whereas wages for study team members providing implementation support were based on median national wages. Fringe benefits were included at a rate of 30%. Salary information was obtained using median national base pay based on position classification and General Schedule level (a United States federal government employee compensation structure).

## Results

**Fig. 1:**
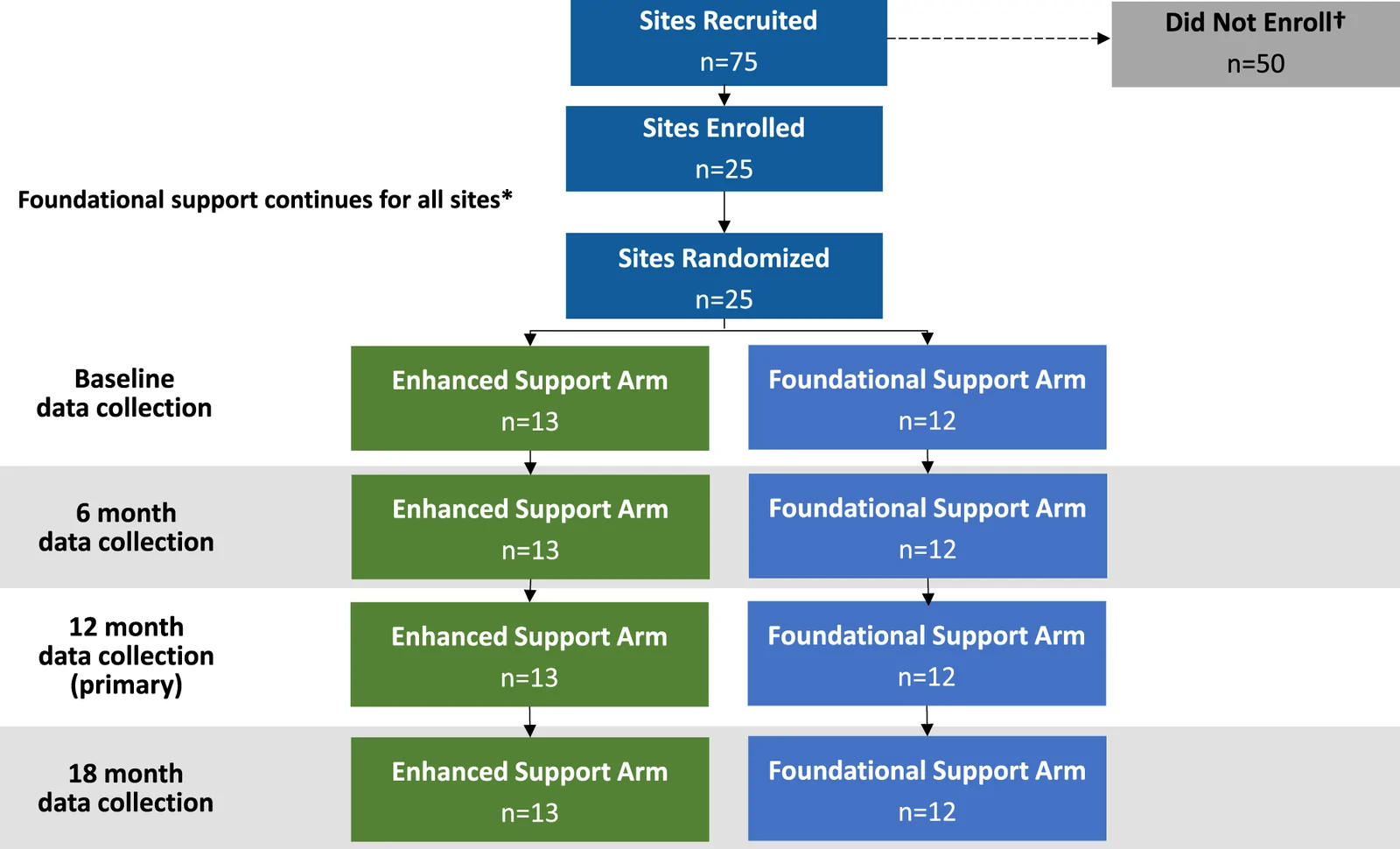
Study consort. legend: *office hour calls end at 12 months and all other foundational supports continue for duration of study. † 31 sites declined: staffing shortages (*n* = 14), no reason (*n* = 12), about to launch program and did not need. implementation support (*n* = 3), leadership did not support participation (*n* = 1); 8 sites not eligible (confirmed trained more than 6 caregivers); 11 sites did not respond to recruitment outreach

Of 75 sites nationally not meeting adoption benchmarks, 25 volunteered and were enrolled and randomized; 12 sites were randomized to foundational arm and 13 sites to enhanced arm. No sites dropped out during the study (Fig. 1).

### Site organizational context (objective 1)

Table 1 presents baseline site characteristics and contextual factors by arm. Participating sites were in the Southern (*n* = 10), Northern (*n* = 7), Midwest (*n* = 5), and Western (*n* = 3) regions of the United States. One-fifth of sites (20%) were rural facilities, with averages slightly higher among foundational sites. Over two-thirds of sites (68%) were classified as low-complexity facilities overall. Fourteen sites (56%) had prior implementation experience with Caregivers FIRST before CSP announced it as a mandatory service (e.g., in fiscal year 2021 or 2022). The mean number of CSP staff for sites was 11, with a slightly higher mean at enhanced sites (12) compared to foundational sites (10). Sites in both arms shared similar organizational context measures for implementation at baseline (ORIC, organizational resilience, and implementation climate scale), and did not influence implementation outcomes.

**Table 1:** Site characteristics and contextual factors by arm

	Overall (*n* = 25)		Enhanced (*n* = 13)		Foundational (*n* = 12)	
Sites with prior implementation of Caregivers FIRST, n (%)^a^	14	(56%)	7	(54%)	7	(58%)
Low facility complexity, n (%)^b^	17	(68%)	9	(67%)	8	(69%)
Rural facility, n (%)^c^	5	(20%)	2	(15%)	3	(25%)
CSP total staffing, mean (SD)^d^	11	(4)	12	(4)	10	(3)
CSP unique applications, mean (SD)^e^	358	(237)	337	(274)	382	(198)
Geographic region, n (%)^f^						
West	3	12%	2	15%	1	8%
Midwest	5	20%	4	31%	1	8%
Northeast	7	28%	4	31%	3	25%
South	10	40%	3	23%	7	58%
Organizational Readiness for Implementing Change [23], mean (SD)^g^	4.5	(0.3)	4.6	(0.2)	4.4	(0.3)
Organizational Resilience [26], mean (SD)^g^	24.5	(2.9)	24.7	(3.3)	24.3	(2.4)
Implementation Climate Scale [27], mean (SD)^g^	2.1	(0.5)	2.1	(0.4)	2.1	(0.6)

*Abbreviations: SD, standard deviation*

*a Collected from site Points of Contact at study start. Classified as prior implementation of Caregivers FIRST (prior to study period) or not*

*b Classified as low complexity (2, 3, and no complexity) versus all others*

*c Rural facility is classified by rural-urban commuting area codes (RUCA) from the VHA Office of Rural Health* [22]

*d Total staffing is the number of full-time equivalent CSP staff hired and planned to hire based on CSP internal programmatic data which was collected 11 months prior to study start*

### Implementation outcomes by implementation support arm (objective 2)

At 12 months, a total of 4,854 unique caregivers of Veterans across the 25 enrolled sites received referrals to CSP caregiver services or support or were enrolled in CSP, with an overall site average of 194 unique referrals (SD = 121). By arm, the site average of referrals or enrollment was 178 (SD = 93) in enhanced versus 212 (SD = 148) in the foundational arm. Across all 25 sites, 307 unique caregivers attended at least one Caregivers FIRST class, and the site percentage of referred or enrolled caregivers attending at least one class ranged from 0.0% to 19.0%. The number of classes offered ranged from 0 to 17 classes. Observed means for penetration and fidelity outcomes were higher in the enhanced arm for all three study timepoints (0–6 months, 0–12 months, and 0–18 months) (Table 2). Almost two-thirds of sites (64%) adopted Caregivers FIRST by 12 months, with 77% in the enhanced arm and 50% in foundational arm (Table 2). By 18 months, 100% of sites in enhanced adopted with 75% in the foundational arm.

**Table 2:** Observed implementation outcomes by arm at 6, 12 (primary), and 18 months (mean (SD); median given unless otherwise specified)

Outcome	Overall (n = 25)	Foundational (n = 12)	Enhanced (n = 13)
**Penetration: Percent of unique caregivers that attended at least one class**			
0–6 months	8.4 (7.1); 7. 9	6.6 (6.5); 5.0	9.9 (7.5); 7.9
0–12 months (primary)	7.2 (4.6); 6.1	6.1 (4.5); 5.6	8.3 (4.5); 7.3
0–18 months	7.2 (4.0); 7.1	6.5 (3.9); 5.3	7.9 (4.0); 7.4
**Penetration: Unique classes offered**			
0–6 months	4.0 (3.0); 4.0	3.1 (2.9); 3.0	4.8 (3.0); 5.0
0–12 months (primary)	7.0 (5.4); 7.0	5.5 (4.9); 4.0	8.4 (5.6); 9.0
0–18 months	10.5 (6.0); 11.0	8.0 (5.1); 6.5	12.9 (6.0); 13.0
**Fidelity: Percent of recommended classes that were delivered by a site**^***a***^			
0–6 months	99.0 (75.1); 100.0	77.1 (72.7); 75.0	119.2 (74.4); 125.0
0–12 months (primary)	87.5 (67.3); 87.5	68.8 (61.4); 50.0	104.8 (70.3); 112.5
0–18 months	87.7 (49.7); 91.7	66.7 (42.0); 54.2	107.1 (49.6); 108.3
**Fidelity: Average number of classes attended per caregiver**			
0–6 months	1.8 (1.2); 2.0	1.6 (1.2); 2.0	2.0 (1.1); 2.2
0–12 months (primary)	2.2 (0.9);2.3	1.9 (1.0); 2.0	2.4 (0.7); 2.6
0–18 months	2.5 (0.6); 2.8	2.5 (0.6); 2.6	2.6 (0.6); 2.8
**Fidelity: Number of unique caregivers who attended at least one ***class*			
0–6 months	7.0 (6.3); 6.0	5.9 (5.8); 5.0	8.1 (6.7); 6.0
0–12 months (primary)	12.3 (8.6); 10.0	11.3 (8.8); 9.5	13.2 (8.6); 10.0
0–18 months	17.6 (9.8); 15.0	16.8 (10.1); 14.5	18.3 (9.8); 17.0
**Adoption: Number of sites meeting minimum of four training classes delivered to at least five caregivers, n(%)**			
0–6 months	14 (56%)	5 (42%)	9 (69%)
0–12 months (primary)	16 (64%)	6 (50%)	10 (77%)
0–18 months	22 (88%)	9 (75%)	13 (100%)

*Abbreviations: SD, Standard Deviation*

*a The percent of recommended classes could exceed 100% if the site offered more than the recommended number during that time period (4 classes in 0–6 months; 8 classes 0–12 months, and 12 classes 0–18 months)*

The estimated mean percentage of caregivers attending a Caregivers FIRST class and number of classes offered was 8.2% and 9.4 classes in the enhanced arm and 6.1% and 5.4 classes in the foundational arm at 12-months. There were no differences in mean percentage of caregivers attending a class (mean difference = 2.2%; 95% CI −1.8%,6.1%; *p* = 0.26) or mean number of classes offered (rate ratio = 1.7; 95% CI 0.9,3.4; *p* = 0.11) in enhanced vs. foundational arms at 12-months. Similarly, no differences between arms in fidelity or adoption outcomes were found at 12-months (Table 3). Similar patterns in estimated rates and differences for both penetration and fidelity outcomes between arms at additional time points were found (Table 3).

**Table 3:** Estimated means by arm and estimated difference in implementation outcomes between enhanced vs. foundational support with associated 95% CI from generalized linear models including stratification variables for 0–6, 0–12 (primary) and 0–18 month time frames

Outcome	Enhanced (n = 13) Estimated Mean (95% CI)	Foundational (n = 12) Estimated Mean (95% CI)	Enhanced vs. Foundational Estimated Difference (95% CI); p-value (12 months)
**Penetration: Percent of unique caregivers that attended at least one class**			
0–6 months	10.5 (6.3,14.8)	7.0 (2.6,11.4)	MD = 3.5 (−2.4, 9.4)
0–12 months (primary)	8.3 (5.4,11.1)	6.1 (3.2,9.1)	MD = 2.2 (−1.8, 6.1); *p* = 0.26
0–18 months	7.7 (5.2,10.1)	6.3 (3.7,8.9)	MD = 1.4 (−2.1, 4.8)
**Penetration: Unique classes offered**			
0–6 months	5.3 (3.4,8.3)	3.0 (1.9,4.8)	RR = 1.8 (0.9, 3.4)
0–12 months (primary)	9.4 (5.8,15.2)	5.4 (3.4,8.8)	RR = 1.7 (0.9, 3.4); p = 0.11
0–18 months	13.7 (10.2,18.4)	8.1 (6.0,11.0)	RR = 1.7 (1.1, 2.6)
**Fidelity: Percent of recommended classes that were delivered by a site**			
0–6 months	128.8 (86.7,170.8)	83.7 (40.3,127.1)	MD = 45.1 (−13.1, 103.3)
0–12 months (primary)	110.4 (70.2,150.6)	72.8 (31.3,114.3)	MD = 37.6 (−18.1, 93.3); p = 0.17
0–18 months	110.4 (81.8,138.9)	69.2 (39.8,98.7)	MD = 41.1 (1.6, 80.6)
**Fidelity: Average number of classes attended per caregiver**			
0–6 months	2.1 (1.5,2.8)	1.6 (1.0,2.3)	MD = 0.5 (−0.4, 1.4)
0–12 months (primary)	2.5 (2.0,3.0)	1.9 (1.3,2.4)	MD = 0.6 (−0.1, 1.4); *p* = 0.10
0–18 months	2.6 (2.2,2.9)	2.4 (2.1,2.8)	MD = 0.2 (−0.3, 0.6)
**Fidelity: Number of unique caregivers whot attended at least one class**			
0–6 months	8.7 (4.9,15.4)	5.4 (3.1,9.6)	RR = 1.6 (0.7, 3.5)
0–12 months (primary)	14.5 (9.6,22.1)	11.1 (7.3,16.8)	RR = 1.3 (0.7, 2.3); p = 0.36
0–18 months	19.1 (14.0,26.0)	17.0 (12.5,23.1)	RR = 1.1 (0.7, 1.7)
**Adoption: Minimum of four training classes delivered to at least five caregivers, n(%)**			
0–12 months (primary)^a^	0.77 (0.48,0.92)	0.50 (0.24,0.77)	OR = 3.3 (0.6, 18.5); p = 0.17

*Abbreviations: MD, Mean Difference; RR, Rate Ratio; OR, Odds Ratio*

*Note: Stratification variables included 1) site complexity (low complexity 1b or 1c versus all others) and 2) prior implementation of Caregivers FIRST (implementation in fiscal year 2021 or 2022 vs. none)*

*a The adoption outcome model does not include stratification variables. Additionally, the primary 0–12 month timepoint was the only one modeled for adoption*

*e CSP unique applications is a proxy for demand for caregiver support services, which is the number of unique applications submitted to the CSP Program of Comprehensive Assistance for Family Caregivers. Number of applications could indicate the demand for caregiver support services at each facility and serve as a proxy for CSP volume or demand. Data collected 11 months prior to study start.*

*f Based on U.S. Census Bureau geographic regions of responding sites (West, Midwest, Northeast, or South) and whether a site was in a rural area as classified by rural–urban commuting area codes (RUCA) from the VHA Office of Rural Health [51, 52]*

*g Site-level means collected from baseline staff surveys. Missing n = 1 site (foundational arm)*

**Fig. 2:**
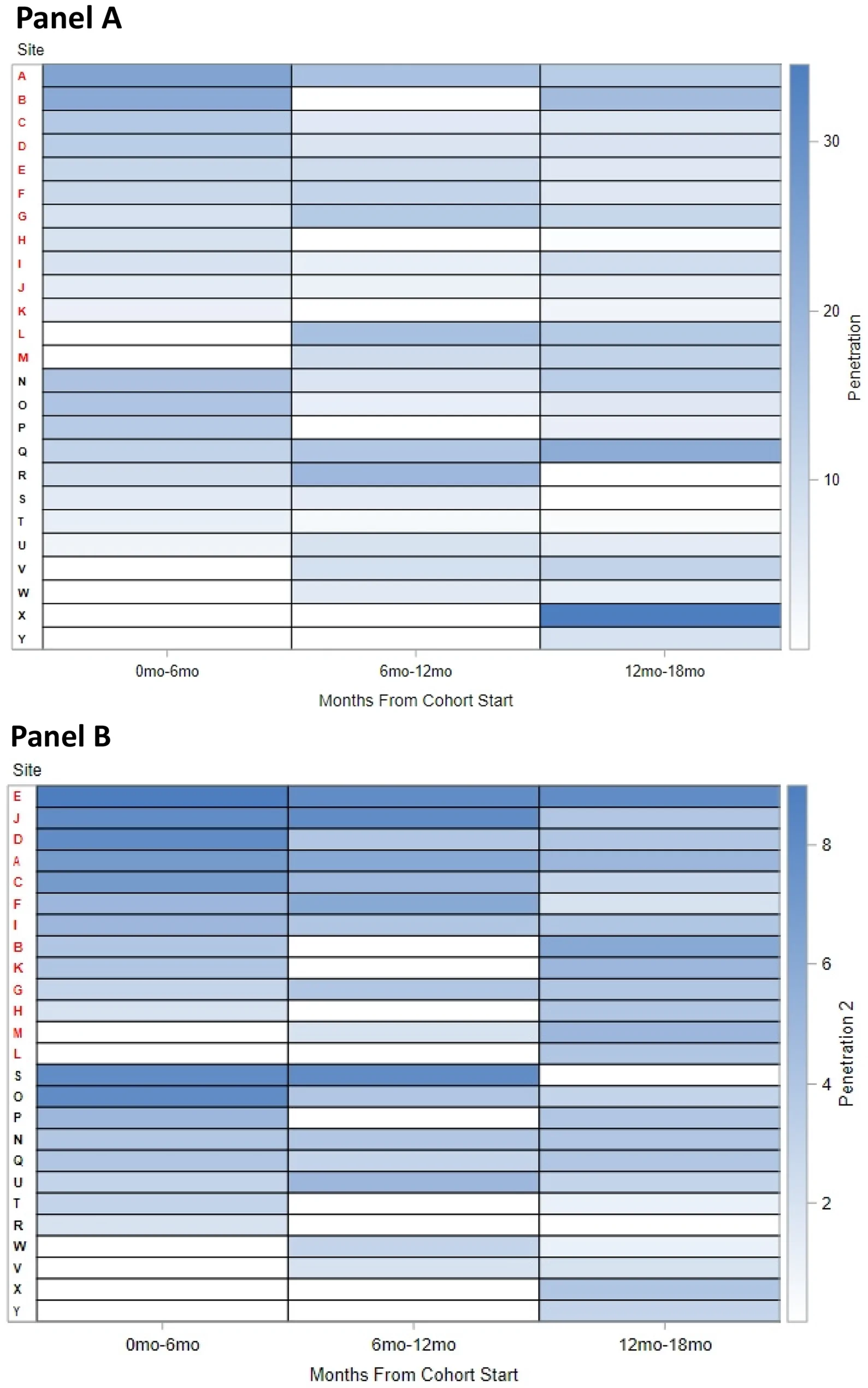
Heat maps of penetration outcomes by site, across study time intervals 0–6 months, 6–12 months, and 12–18 months for sites by arm. legend: letters in red indicate sites in enhanced implementation support arm and letters in black indicate sites in enhanced implementation support arm. Darker blue colors indicate higher penetration. Panel A: the percent caregivers who caregiver attended at least one Caregivers FIRST class; panel B: the number of Caregivers FIRST classes delivered

Heat maps (Fig. 2) display patterns of the penetration co-primary outcome: the percent of caregivers who attended at least one Caregivers FIRST class (panel A), and patterns for the penetration co-primary outcome, number of Caregivers FIRST classes delivered (panel B), across the three six-month time periods of the study (0–6 months, 6–12 months, and 12–18 months) for sites by arm. Within arm, sites are sorted by penetration rate in sequential order of time periods and darker blue colors indicate higher penetration. Both maps illustrate the high variability of penetration by site across time. For percent of caregivers who attended a Caregivers FIRST class, some sites showed stable rates across time (e.g., sites I, J, U), while others started out higher in the first six months and then leveled off or increased in the following time periods (e.g., sites A, B, O decreased, site X increased). For number of Caregivers FIRST classes delivered, sites had higher numbers of classes delivered in the first-time interval and then tapered off (e.g., E, J, D, S, O) with some exceptions (e.g., X, Y). The slightly higher penetration outcomes in the enhanced arm sites (red letters) across time periods compared to foundational arm sites (black letters) were evident by a higher number of blue colored cells compared to white cells.

*Qualitative Context of Implementation Outcomes by Implementation Support Arm (objective 3).* One to four staff at each site completed interviews at baseline, and 1–3 at six months (*n* = 47 total interviews). Thirty-six interviewees were CSP staff working as social workers, nurses, or program support assistants, whereas six were CSP program managers. Others worked as chaplains or social workers from other service lines such as ‘Home Based Primary Care’ or leaders at service line or facility level (*n* = 1–2 for each role).

There were no discernable patterns in terms of implementation outcomes by study arm- the planned points of data triangulation. Staff from foundational and enhanced arms reported similar facilitators and barriers to implementing Caregivers FIRST. Inadequate staffing and caregiver recruitment were the top implementation barriers and the main facilitator was having dedicated staff. Respondents in the enhanced arm could not distinguish between foundational and enhanced support components. Specifically, when asked about the enhanced support calls, staff did not remember them or could not distinguish them from other calls as part of foundational support. One enhanced arm respondent said, “I think more that there was just, it was, it felt a little inefficient because it was like the same thing over and over”. Another respondent from a different site said, “Frankly, I don’t know that I can distinguish between them at this point.”

However, the joint displays revealed qualitative differences in reported implementation experiences by adoption status, regardless of arm. Staff from sites that were able to adopt Caregivers FIRST during the trial reported the use of ‘evaluative and iterative strategies’ [32] while those who continued to be non-adopters did not. Respondents from sites who were able to adopt reported seeking feedback from program participants on how they could improve the program at their site by sending caregivers surveys about Caregivers FIRST (a feedback template was in the foundational support toolkit) and calling past participants to get feedback on how to make the program run more smoothly next time it was offered. Additionally, only respondents from sites able to adopt reported making adaptations to the training curriculum (promoted in the foundational support toolkit), such as adjusting the length, frequency, and modality of classes or offering specific classes for specific types of caregivers (e.g., dementia caregivers). One respondent from an adopting site said, “I decided to do it [Caregivers FIRST] in person because I have low participation for online, anything I do online, so I’ve had higher participation with in-person groups.” Another respondent from an adopting site shared that, “We had done a survey, in general, just to see when they prefer to have classes. So kind of finding that sweet spot of what works for our caregivers’ schedules”.

### Business case analysis (objective 4)

The median cost to provide implementation support over 12 months for the foundational support arm was $14,985 and the enhanced arm was $18,663 (Additional file 6). Personnel costs comprised 97% of total costs in the foundational arm and 94% in the enhanced arm.

## Discussion

Health systems need resource-efficient approaches to scale caregiver training interventions, yet the optimal dose of implementation support remains unclear. This study illuminated the relative value of foundational and enhanced implementation approaches for non-adopter sites to promote penetration and integration of Caregivers FIRST within routine practice, as well as median delivery costs. Regarding our first objective, we found that organizational context including a high readiness to change, resilience ethos, and implementation climate scale were similar in both arms at baseline and did not explain implementation success. Additionally, findings from our second objective revealed that enhanced support was not superior to foundational support at 12-months, although there were higher observed penetration means, fidelity means, and adoption rates across the sites compared to foundational support. In magnitude, implementation outcomes were also higher at 18-months in the enhanced arm, and some met threshold of statistical significance (although without adjustment for multiple testing), which may suggest that an enhanced approach featuring group external facilitation may take additional time to realize an observable benefit to sites seeking to implement a caregiver training intervention. Our third objective yielded insights into common implementation facilitators, barriers, strategies to overcome barriers, and perceptions of implementation support for more information to help interpret any differences in outcomes by study condition. We found that, just as there was no significant difference in implementation outcomes by condition, there were no clear differences in facilitators, barriers or strategies. Staffing and caregiver recruitment were commonly reported challenges across both arms. Certain strategies related to adapting and evaluating the intervention may have been especially useful to support adoption of Caregivers FIRST. The qualitative data also suggest that enhanced implementation support may not have been distinctive enough from the foundational support; this may to some extent explain the null findings. Additionally, enhanced implementation support may not have been sufficiently more intensive than and different from the foundational support; and the fact that foundational support was, in fact, already intense, could to some extent explain our null findings. Overall, receipt of foundational support led to high rates of eventual adoption of the new program. Importantly, the clinical outcomes should not be affected by the lack of differences found in the two implementation strategies but will be evaluated separately by comparing those who participated in the Caregivers FIRST versus similar veterans who did not participate. Finally, our fourth objective identified that implementation activities associated with the enhanced implementation support represented approximately 25% greater median cost when compared to the cost of implementation activities offered as part of foundational support alone.

Our study was powered to detect large effect size differences between arms to warrant the time and effort required to deliver the enhanced support. The approximate effect size differences found in this study were in the 0.50–0.70 range. For example, for the penetration outcome, the percent of caregivers who received referrals to caregiver services and supports and/or were enrolled in CSP, a 1.2 effect size difference that we were powered to detect would translate to a 5.5% difference based on the standard deviation from the trial. Our qualitative findings did not provide additional insight on this finding, because respondents did not have a strong recollection about which activities belonged to the foundational or enhanced support they received, perhaps because all components were delivered in a group). Despite the null finding at 12 months, enhanced support – particularly group external facilitation - may be a cost-efficient approach to improve implementation outcomes over time as larger differences between arms merged at subsequent time points. Enhanced support cost an additional $3,678 per site annually, requiring decision makers to weigh whether this investment justifies modest gains (2% more attending or three additional classes delivered) at their site at 12 months.

Identifying the ‘right dose’ of implementation support required to successfully implement has been a gap in the literature because very few research-driven caregiver programs have been disseminated after study conclusion. As such, there is limited ability with which to define success for scaled pragmatic caregiver trials. We begin to fill this gap by generating initial penetration benchmarks that others might expect in future scaled caregiver trials. In the first rendition of Caregivers FIRST at eight VHA sites, 17% of eligible caregivers received our training [8]. In the second rendition, national scale out evaluated and reported here, approximately 7% of eligible caregivers received training. These two rates are not directly comparable because the denominators defining those ‘eligible for training’ are very different as the referrals for caregiver services and caregiver support were not available in the EHR identifying Veterans with caregivers during the first rendition of Caregivers FIRST. And yet, the rates of penetration, fidelity, and adoption discovered in this study creates an initial range of benchmarks as other teams scale their caregiver interventions in health systems. Benchmarks are particularly important with new CMS policies that allow health systems to be reimbursed for each individual caregiver participating in group caregiver trainings [39, 40]. Success of CMS trainings will depend on the rate of caregivers reached moving forward, because more caregivers in a class means more reimbursement for health systems, creating economies of scale.

In addition, given that the VHA CSP is in the practice of implementing new programs annually as a part of its mission, our findings may be instructive to them and to other operations decisions in health systems. Since a descriptively higher proportion of eligible caregivers were reached with the more costly enhanced implementation support for non-adopter sites, it may be worth the investment to offer targeted intensification of support to sites struggling to adopt caregiver training into routine clinical practices. Unfortunately, we were not able to identify any clear characteristics of sites more likely to benefit from an enhanced approach. As such, health systems with multiple sites could consider deciding based on how much they are able to pay for an enhanced implementation strategy relative to the gain in implementation outcomes reported here and the timeframe under which they need to see trainings completed. By 18 months, 75% of foundational and 100% of enhanced support sites adopted Caregivers FIRST. The foundational support package, consisting of both self-guided tools and interactive resources, allowed most sites to successfully adopt by 18-months even after delays in the first six or 12-month periods after the program mandate. Qualitative interviews at six months indicated that making adaptations to the intervention was practiced among the sites that eventually adopted by 12 and 18-months,underscoring the value of encouraging and allowing sites to make modifications in pragmatic interventions [41].

There are several limitations to this study. Implementation outcomes relied on EHR documentation entered by staff at each site required as part of the data mandate, but may contain missing or incomplete data. Second, we collected qualitative data from a subset of enrolled sites. Although we feel that our qualitative sample provided adequate information to inform our analyses, it is possible that sites not interviewed may have had different implementation experiences. Third, our findings are in the context of 25 VHA sites that were mandated to provide the program and were not able to meet adoption; therefore, findings may not transfer nationally across the VHA system or to other settings. Future research on these outcomes in the absence of a mandate, which could be considered its own implementation strategy, should be explored. Lastly, although this paper does not report clinical outcome data, comparing clinical outcomes will be critical for a deeper understanding of implementation outcomes, and such evaluations are in progress.

In general, this study provides real-world evidence of the effort needed to implement caregiver trainings at scale, which is timely as the 2022 National Caregiver Strategy to Support Family Caregivers [42] include spreading evidence-based trainings as a tenet of its recommendations and CMS has initiated several initiatives to explicitly support caregivers of persons with dementia or patients who could benefit therapeutically from their caregiver being trained. Given the topics covered in Caregivers FIRST, this training would be an appropriate candidate for consideration for health systems and providers since it covers the basics of daily care, home safety, safe transfers, how to preserve Veteran independence and avoid injury, communication techniques, and problem solving.

## Conclusions

This study helps identify effective elements and dose of implementation support for efficient scaling of future caregiver training programs. By 18 months, 75% of foundational and 100% of enhanced support sites adopted Caregivers FIRST. Investing in foundational or enhanced support targeting non-adopter sites could improve integration of caregiver skills training into routine clinical care [43].

## Electronic supplementary material

Below is the link to the electronic supplementary material.

Supplementary material 1

Supplementary material 2

Supplementary material 3

Supplementary material 4

Supplementary material 5

Supplementary material 6

## Data Availability

A de-identified version of the datasets used and/or analyzed for the current study is available from the corresponding author on reasonable request.

## Abbreviations

BIA: Business Case Analysis
Caregivers FIRST: Caregivers Finding Important Resources, Support, and Training
CI: Confidence Interval
CMS: Centers for Medicare and Medicaid Services
CSP: Caregiver Support Program
EHR: Electronic Health Record
ERIC: Expert Recommendations for Implementing Change typology
Function QUERI: Optimizing Function and Independence Quality Enhancement Research Initiative
GUIDE Model: Guiding an Improved Dementia Experience
MD: Mean Difference
OR: Odds Ratio
ORIC: Organizational Readiness to Implement Change
REDCap: Research Electronic Data Capture
REP: Replicating Effective Programs
RR: Rate Ratio
RUCA: Rural-Urban Commuting Area codes
SD: Standard Deviation
StaRI: Standards for Reporting Implementation Studies
U.S.: United States
VHA: Veterans Health Administration
