# Supplementary material for: Evaluating two implementation approaches to support a nationally mandated caregiver skills training in veterans affairs: a type III hybrid implementation-effectiveness randomized controlled trial
Source: Implement Sci Commun. 2026 Jun 11;7:166. doi: 10.1186/s43058-026-00960-9 (PMC13508206; doi:10.1186/s43058-026-00960-9)
Supplement: Supplementary file 1 [file 43058_2026_960_MOESM1_ESM.docx]

**Additional file 6. Time and cost estimates of implementation strategy bundle (Median(IQR))**

|  | ***0-3 months*** | | ***4-6 months*** | | ***7-12 months*** | | ***0-12 months*** | | |
| --- | --- | --- | --- | --- | --- | --- | --- | --- | --- |
|  | **Hours** | **Cost ($)** | **Hours** | **Cost ($)** | **Hours** | **Cost ($)** | **Median hours for 12 months** | **Median hours for 12 months** | **Median cost for 12 months ($)** |
| **Foundational (n=12)** | | | | | | | | | |
| ***Site-level*** | | | | | | | | | |
| **Office hours^a^** | 5 | 312 | 2 | 128 | 4 | 246 | 10 | **258** (199-368) | **14465** (11866-23464) |
|  | (4-6) | (261-385) | (1-3) | (61-188) | (3-7) | (183-401) | (8-15) |  |  |
| **Site reported effort** | 65 | 3931 | 26 | 1608 | 130 | 8015 | 247 |  |  |
|  | (39-120) | (2544-7488) | (23-39) | (1127-2432) | (98-189) | (5939-10492) | (189-349) |  |  |
| ***External*** | | | | | | | | | |
| **Office hours^a^** | 3 | 139 | 3 | 139 | 7 | 241 | 13 | **13** | **520** |
|  | (3-3) | (133-146) | (3-3) | (133-146) | (7-7) | (184-298) | (13-13) | (13-13) | (449-590) |
| **Total Foundational** |  | | | | | | | | **14985** |
| **Enhanced (n=13)** | | | | | | | | | |
| *Site-level* | | | | | | | | | |
| **Office hours^a^** | 3 | 206 | 2 | 122 | 5 | 305 | 9 | **287** (198-305) | **17512** (12060-18945) |
|  | (3-6) | (183-388) | (0-3) | (0-183) | (1-6) | (61-365) | (6-18) |  |  |
| **Site reported effort** | 59 | 3563 | 20 | 1224 | 169 | 10294 | 267 |  |  |
|  | (46-98) | (2771-5939) | (13-39) | (792-2375) | (52-208) | (3167-12669) | (195-293) |  |  |
| **Group External facilitation** | - | - | 4 | 244 | - | - | 4 |  |  |
|  |  |  | (3-5) | (183-328) |  |  | (3-5) |  |  |
| *External* | | | | | | | | | |
| **Office hours^a^** | 3 | 139 | 3 | 139 | 7 | 241 | 13 | **13** | **520** |
|  | (3-3) | (133-146) | (3-3) | (133-146) | (7-7) | (184-298) | (13-13) | (13-13) | (449-590) |
| **Group External facilitation** | - | - | 12 | 631 | - | - | 12 | **12** | **631** |
|  |  |  | (12-12) | (573-688) |  |  | (12-12) | (12-12) | (573-688) |
| **Total Enhanced** |  | | | | | | | | **18663** |

*a Office Hours was provided monthly using Microsoft Teams to both foundational and enhanced arms via holding virtual Office Hours which were facilitated discussions on Caregivers FIRST implementation, highlight best practices, and promote access to electronic implementation resources and materials, and facilitating cross-site communication and problem solving. Hours of time cost was calculated based on site-level attendance and personnel resources. Since Office Hours were offered by external research team personnel to sites regardless of randomization arm, they are included in both strategy bundle cost estimate.*

*NOTE: ‘External’ facilitation cost estimates refers to personnel costs associated with Durham VA Function QUERI staff and infrastructure for providing foundational and enhanced implementation support. Wages based on mid-level VA social worker annual salary and program coordinator.*
