# Supplementary material for: Evaluating two implementation approaches to support a nationally mandated caregiver skills training in veterans affairs: a type III hybrid implementation-effectiveness randomized controlled trial
Source: Implement Sci Commun. 2026 Jun 11;7:166. doi: 10.1186/s43058-026-00960-9 (PMC13508206; doi:10.1186/s43058-026-00960-9)
Supplement: Supplementary file 3 [file 43058_2026_960_MOESM3_ESM.docx]

**Additional file 2. Staff Implementation Survey Consort (Baseline)**

**Points of Contact sent electronic survey**

**n = 127, Sites: n = 25**

**Responded to survey**

**n = 72, Sites: n = 24	(min per site = 1, max per site = 6)**

(Partial Response: n = 10, Complete Response n = 62)

**Analyzed**

**n = 72**

Sites n = 24

**Excluded: n = 10**

(Left VA position: n = 1, Not on Staff Listing n = 9)

**Excluded: n = 55**

(no response: n = 54, not involved in CGF: n = 1)
