# Supplementary material for: Evaluating two implementation approaches to support a nationally mandated caregiver skills training in veterans affairs: a type III hybrid implementation-effectiveness randomized controlled trial
Source: Implement Sci Commun. 2026 Jun 11;7:166. doi: 10.1186/s43058-026-00960-9 (PMC13508206; doi:10.1186/s43058-026-00960-9)
Supplement: Supplementary file 5 [file 43058_2026_960_MOESM5_ESM.docx]

**Additional file 4. Site characteristics and contextual factors of qualitative sample**

|  | Total  (n = 25) | | Qualitative sample  (n = 12) | |
| --- | --- | --- | --- | --- |
|  |  |  |  |  |
| Implementation arm, n(%)  foundational  enhanced | 12  13 | (48%)  (52%) | 3  9 | (25%)  (75%) |
| Sites with prior implementation of Caregivers FIRST, n (%) ^a^ | 14 | (56%) | 7 | (58%) |
| Low facility complexity, n (%)^b^ | 17 | (68%) | 9 | (75%) |
| Rural facility, n (%)^c^ | 5 | (20%) | 2 | (17%) |
| CSP total staffing, mean (SD)^d^ | 11 | (4) | 11 | (4) |
| CSP unique applications, mean (SD) ^e^ | 358 | (237) | 303 | (245) |
| Geographic region, n (%)^f^ |  |  |  |  |
| West | 3 | 12% | 3 | 25% |
| Midwest | 5 | 20% | 3 | 25% |
| Northeast | 7 | 28% | 3 | 25% |
| South | 10 | 40% | 3 | 25% |

*Abbreviations: SD, standard deviation.*

*a Collected from site Points of Contact at study start.* *Classified as prior implementation of Caregivers FIRST (prior to study period) or not.*

*b Classified as low complexity (2, 3, and no complexity) versus all others.*

*c Rural facility is classified by rural-urban commuting area codes (RUCA) from the VHA Office of Rural Health*

*d Total staffing is the number of full-time equivalent CSP staff hired and planned to hire based on CSP internal programmatic data which was collected 11 months prior to study start.*

*e CSP unique applications is a proxy for demand for caregiver support services, which is the number of unique applications submitted to the CSP Program of Comprehensive Assistance for Family Caregivers. Number of applications could indicate the demand for caregiver support services at each facility and serve as a proxy for CSP volume or demand. Data collected 11 months prior to study start.*

*f* *Based on US Census Bureau geographic regions of responding sites (West, Midwest, Northeast, or South) and whether a site was in a rural area as classified by rural–urban commuting area codes (RUCA) from the VHA Office of Rural Health.*
