# Supplementary material for: Evaluating two implementation approaches to support a nationally mandated caregiver skills training in veterans affairs: a type III hybrid implementation-effectiveness randomized controlled trial
Source: Implement Sci Commun. 2026 Jun 11;7:166. doi: 10.1186/s43058-026-00960-9 (PMC13508206; doi:10.1186/s43058-026-00960-9)
Supplement: Supplementary file 6 [file 43058_2026_960_MOESM4_ESM.docx]

# **Additional file 3. Staff Cost Estimate Survey Consort**
